# Biopreservation of Food Using Bacteriocins From Lactic Acid Bacteria: Classification, Mechanisms, and Commercial Applications

**DOI:** 10.1155/ijm/8723968

**Published:** 2024-11-28

**Authors:** Dhea Alya Putri, Jiang Lei, Nia Rossiana, Yolani Syaputri

**Affiliations:** ^1^Department of Biology, Faculty of Mathematics and Natural Sciences, Universitas Padjadjaran, Sumedang Regency, West Java, Indonesia; ^2^School of Inspection and Testing Certification, Changzhou Vocational Institute of Engineering, Changzhou 213164, China; ^3^Center for Bioprospection of Natural Fibers and Biological Resources, Faculty of Mathematics and Natural Sciences, Universitas Padjadjaran, Sumedang Regency, West Java, Indonesia

**Keywords:** bacteriocin, biopreservative, foodborne disease, lactic acid bacteria

## Abstract

Food is the primary substance needed by humans to survive. However, food is easily contaminated by spoilage bacteria, which cause a decrease in quality and shelf life. Moreover, spoilage bacteria in food can be pathogenic, leading to foodborne disease that endangers human health. This issue has also driven the widespread use of synthetic preservatives, which have negative effects both in the short and long term. Biopreservation efforts utilizing bacteriocins produced by lactic acid bacteria offer an alternative solution to prevent spoilage and extend the shelf life of food. These bacteriocins are safe to use as they are produced by lactic acid bacteria that are approved for use in food. The application of various types of bacteriocins as biopreservatives has been widely conducted. Several other types of bacteriocins are continuously being researched and developed to ensure their safety and suitability for use as food biopreservatives. This article highlights bacteriocins, including their classification, general overview, mechanisms of action, differences from antibiotics, diversity, applications, prospects, and challenges as future food biopreservatives. Additionally, this article presents commercial bacteriocins, namely, nisin and pediocin, which are frequently used for food preservation.

## 1. Introduction

Foodborne diseases are a global issue requiring special attention. In countries with poor food safety monitoring systems, cases of food poisoning have increased, leading to deaths [1]. The diseases affect individuals across various age groups, specifically children under five and low-income communities [2]. This is associated with the consumption of contaminated meat, canned food, dairy products, fermented products, and poultry, particularly by pathogenic bacteria [3]. Common pathogenic bacteria contaminating food include *Escherichia coli*, *Salmonella* sp., and *Staphylococcus aureus* in meat [4]; *Enterobacter aerogenes*, *Listeria monocytogenes*, *Pseudomonas* sp., *Shewanella* sp., *Vibrio* sp., and *Yersinia* sp. in seafood [5]; *Bacillus anthracis*, *Bacillus cereus*, *Clostridium botulinum*, and *Pseudomonas aeruginosa* in dairy [6]; *Enterobacter* sp., *Shigella* sp., and *Staphylococcus* sp. in fruits [7]; and *Erwinia carotovora*, *Clavibacter michiganensis*, *Pseudomonas syringae*, *Ralstonia solanacearum*, and *Xanthomonas campestris* in vegetables [8].

Spoilage or pathogenic bacteria can pose dangers to food safety and human health [9]. These bacteria cause changes in aroma, color, and taste; gas and slime production; and a decrease in food pH, making it unfit for consumption [10]. The industrial sector, especially the food industry, is also affected by food contamination during processing, transportation, and storage [11]. *Bacillus coagulans* causes significant economic losses because it can lead to acidification and unpleasant tastes in canned vegetable products [9]. *Salmonella* sp. contaminates poultry and meat products, which can cause salmonellosis. The European Centre for Disease Prevention and Control (ECDC) reported that *Salmonella* sp. was responsible for the highest number of human infections and caused illness in 91,857 people in the European Union in 2018. Hospitalizations due to salmonellosis were reported to reach 16,816 cases. Additionally, the economic losses, including medical costs, premature death, and lost wages and productivity, can exceed 3 billion per year [12].

Traditional and modern preservation techniques have been extensively used to control the growth of spoilage bacteria [2] and maintain the quality, physicochemical properties, safety, and stability of food [13]. Synthetic chemical preservatives are commonly used to extend the shelf life of food. Long-term consumption of these preservatives has negative effects on the body [14]. Sodium benzoate, added to carbonated drinks, pickles, and sauces, can worsen asthma. Its neurotoxins and carcinogenic compounds can cause fetal abnormalities [15]. Nitrites, which combine with hemoglobin to form methemoglobin, can result in blue baby syndrome. Nitrites are also carcinogenic if consumed in excessive amounts. Parabens have been reported to disrupt the hormonal system and physiological functions of the body [11]. This has driven the development of natural preservation efforts, also known as sustainable biopreservation, to address the harmful effects of synthetic preservatives.

As a natural preservation method, biopreservation is trending to prevent spoilage and enhance food safety. This method includes using microbes, such as lactic acid bacteria and related products. Lactic acid bacteria are a group of Gram-positive, nonmotile, non-spore-forming [14], facultative anaerobes, and rod or cocci-shaped bacteria [16]. These microorganisms are found in dairy, fruits [17], vegetables, meat, gastrointestinal and urogenital tracts, soil, and water [18]. The bacteria are starter cultures in food fermentation processes (K. N.) [19] and have been classified as Generally Recognized as Safe (GRAS) with a Qualified Presumption of Safety (QPS) status [20, 21]. In addition, the microorganisms are known as probiotics with therapeutic properties to improve human health. In combating pathogenic microbes, lactic acid bacteria create a competitive environment, compete for nutrients, modulate target cell immunity, and produce antimicrobial compounds such as acetic acid, propionic acid, exopolysaccharides [18], lactic acid, hydrogen peroxide, bacteriocin [22], carbon dioxide [23], diacetyl, ethanol, and reuterin [1]. Some genera of lactic acid bacteria include *Aerococcus*, *Alloiococcus*, *Carnobacterium*, *Dolosigranulum*, *Enterococcus*, *Lactobacillus*, *Lactococcus*, *Lactosphaera*, *Melissococcus*, *Tetragenococcus*, *Vagococcus* [17], *Globicatella*, *Weissella* [24], *Leuconostoc*, *Oenococcus*, *Pediococcus*, and *Streptococcus* [20].

The high demand for safe and natural preservatives opens new research opportunities to uncover different efficient bacteriocins against foodborne pathogens [25]. In this context, bacteriocin is a ribosomally synthesized bioactive peptide or protein with antimicrobial activity against bacteria, fungi, viruses, and parasites [26]. The uniqueness of bacteriocins lies in their ability to combat opportunistic pathogenic bacteria, including drug-resistant species [17], stable at acidic to neutral pH and high temperatures [19], easily degraded by proteolytic enzymes in the human digestive tract [26], and nontoxic [27]. These odorless, colorless, and tasteless molecules [26] can enhance the shelf life, nutrition, and sensory characteristics of food [2]. Therefore, bacteriocin presents a promising potential as a future food biopreservative agent to replace long-used synthetic preservatives.

This research aims to provide general information about bacteriocin, including classification, overview, mechanisms of action, diversity, and application as food biopreservatives. The differences between bacteriocin and antibiotic are also reported, while nisin and pediocin are reviewed for food preservation. The prospects and advancements of the research are emphasized in the development and application of bacteriocin as future food biopreservatives.

## 2. Classification of Bacteriocin

Bacteriocin is produced by lactic acid bacteria in Gram-positive and Gram-negative groups [17, 19]. This bioactive peptide is produced as part of self-defense mechanism against the environment [28]. Bacteriocin-producing bacteria are resistant to the compounds they produce. This occurs because these bacteria produce immunity proteins [17] or use efflux pumps to protect themselves from damage caused by their own bacteriocins [29]. Based on the structure, physicochemical properties, and molecular characteristics, bacteriocin is classified into five main classes as follows.

### 2.1. Class I Bacteriocin

Class I bacteriocin contains 19 to 50 amino acids [25], has low molecular mass (< 5 kDa) and posttranslational modifications, and is heat-stable [3]. This peptide is globular or linear, containing *β*-methyllanthionine, modified lanthionine amino acids, and dehydrated amino acids collectively known as lantibiotic [1, 14]. Similarly, Class I bacteriocin is divided into four subclasses as follows:

a. Subclass Ia Bacteriocin
  Subclass Ia bacteriocin consists of Type A lantibiotic with an elongated or linear structure, positive charge, and a size of 2–4 kDa [30]. In this subclass, the peptide is flexible and hydrophobic [17], containing lanthionine and *β*-methyllanthionine. Examples of Subclass Ia bacteriocin include ericin A and S, subtilin [30], epidermine, and nisin [26].
b. Subclass Ib Bacteriocin
  Subclass Ib or labyrinthopeptin is nonflexible [17] globular Type B peptide with a negative or no charge, and a size of 2 to 3 kDa [30]. In addition, the bacteriocin contains labyrinthine and labionin. Examples of the subclass include mersacidin, paenibacillin, sublancin 168 [30], actagardine, amylolysin, michiganin A, pseudomycoicidin [31], and labyrinthopeptin A1 [25].
c. Subclass Ic Bacteriocin
  Subclass Ic bacteriocin or sanctibiotic consists of lantibiotic with two components [30], namely, sulfur and alpha carbon. Examples include thuricin CD [25], haloduracin, lichenicidin [30], geobacillin II, and formicin [31].
d. Subclass Id Bacteriocin
  Subclass Id bacteriocin consists of cyclic peptide, and a typical example is subtilosin A [30].

### 2.2. Class II Bacteriocin

Class II or nonlantibiotic is small (< 10 kDa) and flexible bacteriocin [17]. This peptide contains 30 to 60 amino acids; is positively charged; lacks lanthionine [3], not posttranslationally modified [13], hydrophobic; and shows good stability against high temperatures and a wide pH range [2]. The class features disulfide bridges between two cysteines, contributing to antimicrobial activity and heat resistance [32]. In addition, the bacteriocin lacks lanthionine and possesses an amphiphilic helical structure for facilitating entry into the target microbial cell membrane, leading to depolarization and cell death [26]. This peptide has a narrow bactericidal activity spectrum [25] and is divided into four subclasses as follows:

a. Bacteriocin Subclass IIa
  Subclass IIa is pediocin-like bacteriocin with highly conserved hydrophilic regions [2] and a YGNGV-C consensus sequence at N-terminal [14]. This bacteriocin is small, heat-resistant [25], and contains 35 to 50 amino acids [17]. Moreover, the peptide is active against *Listeria monocytogenes* [17, 26]. Examples include enterocin [10, 26]; bavaricin MN, curvacin A, and lactococcin A and B [18]; leucocin A [13]; B-TA1 Ia and pediocin PA-1/AcH [16]; coagulin SRCAM 37, 602, and 1580 [30]; sakacin A and P; and leucocin A [25].
b. Bacteriocin Subclass IIb
  Subclass IIb is an unmodified two-peptide bacteriocin that works synergistically to produce an antimicrobial effect [26, 30]. This peptide shows high sequence homology [10] and is heterodimeric [13]. Examples include bacthuricin F4, cerein MRX1, and thuricin H, S, and 17 [30]; enterocin X [33]; lactocin G [34] and 705 [13]; plantaricin A [33], EF, and JK [25]; lactococcin F, G [16], and M [18]; and lacticin F and 3147 [26].
c. Bacteriocin Subclass IIc
  Subclass IIc is circular bacteriocin containing 35 to 70 amino acids, positively charged, resistant to proteolytic enzymes, as well as classified into cystibiotics and thiolbiotics [17]. The bacteriocin contains closed-loop cyclic peptide [10], where the N- and C-terminals are connected by covalent bonds [18]. Examples include carnocyclin A, circularin A, gassericin A, lactococcin [26], cerein 7A and 7B, lichenin, thuricin 439 [30], lactococcin B [16], reutericin 6 [13], lactocin B [34], enterocin AS-48, and garvicin ML [25].
d. Bacteriocin Subclass IId
  Subclass IId is linear and single bacteriocin [17] with unmodified molecular structures [2]. Examples include lichenin, cerein 7A and 7B, thuricin 439 [30], lacticin Q, and aureocin A53 [35]. As typical members of the subclass, bactofencin and LsbB are highly cationic and resemble some antimicrobial peptide in eukaryotes [25].

### 2.3. Class III Bacteriocin

Class III bacteriocin (bacteriolysin) is an unmodified peptide [17], with large molecular weights (> 30 kDa), and heat-sensitive [36]. At high temperatures (100°C), this bacteriocin becomes inactive in 30 min [1]. Class III bacteriocin is divided into Subclass IIIa (bacteriolysin), IIIb (nonlytic), and IIIc (tailocin) [33]. Bacteriolysin is a large lytic polypeptide (27–35 kDa) that is heat-sensitive and targets the peptidoglycan layer [37]. Examples include enterolysin A, lysostaphin, millericin B, and zoocin A. Meanwhile, nonlytic bacteriocin is a large polypeptide known for decreasing glucose uptake. Examples include casecin 80, helveticin J [38], and dysgalacticin. Tailocin is a multiprotein complex targeting lipopolysaccharides and resembling phage tails, with molecular weights of 20–100 kDa. Examples comprise diffocin and monocin [37]; colicin, klebicin, helveticin I, megacin A-216, and A-19213 [30]; NCPP32355 and SW1-1 [31]; helveticin V-1829 and lactacin A and B [14]; and helveticin M [25].

### 2.4. Class IV Bacteriocin

Class IV bacteriocin is a large complex consisting of chemical groups [36], such as carbohydrate, protein, and lipid molecules [3]. This peptide inhibits the growth of both Gram-positive and Gram-negative bacteria [10] due to the complex structure. Examples include glycocin F [13], pediocin SJ-1 [18], lactocin 27, leuconocin S, and plantaricin S [16]. Additionally, bacteriocin produced by Gram-negative bacteria, such as colicin and microcin, is categorized in this class due to microbial targets, protein size, and antigenicity or immune mechanism [13].

Colicin is a peptide with a high molecular mass of 30 to 80 kDa. The peptide is produced by *Escherichia coli* containing colicinogenic plasmids. Meanwhile, microcin is a low molecular mass (1–10 kDa) and highly stable bacteriocin. The characteristics include resistance to protease enzymes, extreme pH, and temperature. The peptide is produced by enteric bacteria under stressful conditions, specifically during nutrient deficiency [26].

### 2.5. Class V Bacteriocin

Class V bacteriocin is circular and belongs to a cyclic peptide with bonds from head to tail along the backbone [31, 39]. Cyclization is the formation of peptide bonds between N and C termini of the prepeptide through enzymatic reactions. This bacteriocin is not subjected to posttranslational modifications from head to tail but shows high thermal stability and isoelectric points. An example of a Class V bacteriocin is amylocyclicin produced by *Bacillus amyloliquefaciens* FZB42 [31].

## 3. General Overview of Bacteriocin as Food Biopreservatives

Bacteriocins are antimicrobial peptides or proteins produced by bacteria, particularly lactic acid bacteria [29]. Bacteriocins are produced extracellularly during the late exponential to early stationary growth phase [17] and are secreted into the environment at subinhibitory concentrations [40]. These peptides are synthesized by coding genes located on plasmids or chromosomes as part of the bacteriocin coding genes, immunity protein genes, and lysis genes involved in the release of bacteriocins from bacterial cells [14]. Based on their characteristics, bacteriocins are cationic molecules that are either hydrophobic or amphiphilic [33]. Bacteriocins have various benefits, including use as probiotics; food preservatives; plant growth promoters; treatments for oral, dental, and skin diseases; anticancer therapies; and health care for both humans and livestock [29].

In the food industry, bacteriocins serve as natural additives to extend the shelf life of food [29]. Additionally, bacteriocins can improve food quality and sensory properties, such as preventing spoilage in cheese and enhancing its rate of proteolysis [26]. Bacteriocins are chosen as food biopreservatives due to their stability across a wide range of pH and temperatures [28]. These compounds are tasteless, colorless, and odorless and can easily penetrate the food matrix [29]. Moreover, bacteriocins are considered safer than chemical preservatives, which can have negative effects [14].

The effectiveness of bacteriocins in food preservation is influenced by several factors, including protein and lipid content, pH, manufacturing processes, additives, enzymatic degradation [2, 28], storage temperature [39], and the interactions and characteristics of spoilage bacteria [10]. Before being applied to food, bacteriocins need to be extracted using various methods such as dialysis membranes, pH-mediated cell adsorption or desorption, macroporous resin columns, ammonium sulfate precipitation, solvent extraction, and chromatography [33].

The application of bacteriocins as biopreservatives has been widely implemented to prevent spoilage in various food products [28]. Food products commonly preserved with bacteriocins include eggs, milk, meat, vegetables [26], and processed food products (cheese and sausage) [2]. Another application of bacteriocins is as bioactive food packaging [26]. Biofilms are often found on and formed around food. Biofilms are collections of one or more types of bacteria that can attach to solid surfaces (food industry equipment, transportation, etc.) and food (fruits, vegetables, meat, bones, etc.). These biofilms consist of extracellular matrices made up of polysaccharides, cellulose, and proteins or exogenous DNA. Biofilms function to protect bacterial cells by exhibiting antimicrobial properties and resistance to drying, disinfection, chemicals, and fluid flow in pipes. The formation of biofilms can result in contaminated food that is unsafe for consumption [41]. Food packaging induced with bacteriocins is a solution to protect food surfaces from bacteria, ensuring food safety and shelf life [10].

Bacteriocin is added as an additional ingredient in food or active packaging in pure or semipure forms. The bacterial cultures can also be added as starters to ferment food [13] or produce bacteriocin in situ [26]. Additionally, the peptide can be combined with other antimicrobial compounds, such as essential oils, cinnamon, and oregano, to reduce the excessive use of bacteriocin [25]. Chelating agents, including EDTA, are combined with bacteriocin to produce a stronger antimicrobial effect. Clinically, EDTA has shown significant antimicrobial properties against pathogenic microbes [39]. EDTA can bind magnesium ions in the lipopolysaccharide layer, disrupting the outer layer of the target bacteria. Combining bacteriocins with nonthermal techniques, such as hydrostatic pressure (HP) and pulsed electric field (PEF), can also enhance the inactivation of target bacteria [41].

## 4. Mechanism of Action of Bacteriocin as Antimicrobials

Bacteriocins are generally narrow-spectrum [1] and exhibit bacteriostatic and bactericidal effects on bacterial species closely related to the producing strain [14, 17]. These properties determine whether the bacteria are killed or if the log phase of growth is extended [29]. Bacteriostatic and bactericidal activity may occur with or without cell lysis [42]. At concentrations higher than natural levels, the peptide reports very strong antimicrobial activity [40] against pathogenic bacteria, including Gram-positive and Gram-negative bacteria, such as *Micrococcus* sp., *Shigella* sp., *Streptococcus* sp., *Vibrio* sp. [3], *Bacillus cereus*, *Campylobacter jejuni*, *Clostridium tyrobutyricum*, *Escherichia coli*, *Helicobacter pylori* NCIPD 230, *Listeria innocua*, *Listeria monocytogenes*, *Salmonella typhimurium*, and *Staphylococcus aureus* [28].

In general, bacteriocins have specific molecular mechanisms to combat target microbes. These mechanisms include pore formation, degradation of cellular DNA, disruption caused by specific cleavage of 16S rDNA, inhibition of peptidoglycan synthesis [43, 44], autolytic enzyme activity, and bacterial spore formation. Bacteriocins diffuse into the target microbial cell and react with the peptidoglycan precursor, lipid II, which acts as an anchor molecule [29]. These peptides bind to specific receptors on the membrane or cell wall of the target microbe, such as lipoteichoic acid and teichoic acid, causing instability within [16]. This leads to the formation of pores in the microbial cell membrane, increasing membrane permeability, disrupting proton motive force (PMF), and causing the loss of intracellular materials [45]. At this stage, small molecules such as amino acids, ATP, and ions like phosphorus, potassium, and magnesium move to the external environment of the target microbial cell through the formed pores. The pH gradient and membrane potential are also disrupted [29]. Furthermore, the disruption of PMF inhibits cell growth and energy production, leading to leakage of the target microbial cell components [11]. In addition to pore formation, bacteriocins can inhibit cell proliferation or division [46] and the synthesis of DNA, RNA, and proteins in target microbial cells [11]. DNA, RNA, and protein synthesis are inhibited when there is a deficit of ions and ATP within the target microbial cell. ATP levels decrease because it cannot be produced due to phosphate leakage from the target microbial cell, and ATP consumption increases to maintain electrical potential [29].

The main bacteriocin receptors are located in the cytoplasm and include anionic lipid molecules [34]. This anionic nature makes them targets for cationic bacteriocins [25]. The cationic nature of bacteriocins arises from lysine and arginine residues, which consist of 12–45 amino acids [45]. The interaction of bacteriocins with target microbes occurs in two stages. In the first stage, a reversible interaction occurs involving the physical adsorption of bacteriocins into the cell via the receptor. Removing bacteriocins at this stage does not result in damage to the target microbial cell, as no permanent physiological damage has occurred. In the second stage, irreversible pathological changes occur due to specific biochemical damage to the target microbial cell. Some bacteriocins require docking molecules, such as mannose permease and lipid II, to interact with the target microbial cell membrane. Meanwhile, other bacteriocins do not require docking molecules. Microbial sensitivity to bacteriocins is due to their interaction with the surface and membrane of the target microbial cell [33].

**Figure 1:**
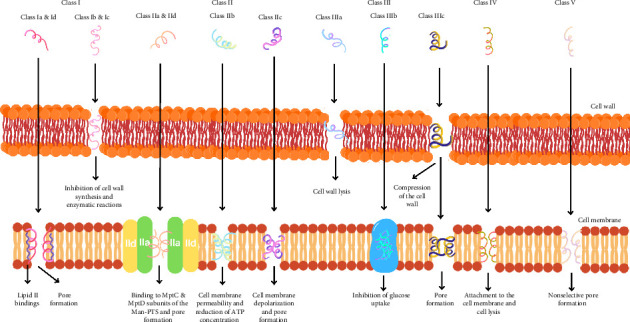
Mechanism of action of bacteriocin as antimicrobials.

Each bacteriocin has a specific mode of action depending on its primary structure, physicochemical characteristics, posttranslational modifications, the producing bacterial strain, and dosage used [29]. These mechanisms include those that focus on the cell membrane or adsorption on the surface of the target microbial cell, those that are active within the cell (cell lysis), and those that inhibit protein synthesis and gene expression [17, 26, 45]. In cell lysis, bacteriocins interact with cell membrane components such as teichoic acid, lipoteichoic acid, and teichuronic acid. Autolytic enzymes associated with acids are released and activated, resulting in cell autolysis [29]. Bacteriocins can also form septa to inactivate the target microbe [42]. The antimicrobial mechanism of bacteriocins in various classes is shown in Figure 1.

Subclass Ia and Id bacteriocins work on the cytoplasmic membrane by forming pores [13, 47]. This pore formation depends on lipid II receptors and peptidoglycan, which serve as docking molecules [34]. The mechanism of Subclass Ib and Ic bacteriocins involves disrupting cell wall biosynthesis and enzymatic reactions [13, 47]. Electrostatic forces help bacteriocins bind to the anionic phospholipid membrane of the target microbial cell. Connections are formed by the N-terminal domain through linking to pyrophosphate II in the cell membrane's lipid. This results in inhibited peptidoglycan synthesis and increased membrane permeability, facilitating pore formation and enabling ion efflux from the target microbial cell. Furthermore, neutralization reactions between positively charged molecules and negatively charged phospholipids lead to cell death [41].

Subclass IIa and IId bacteriocins bind to MptC and MptD subunits on mannose phosphotransferase permease (Man-PTS), opening intrinsic channels. These bacteriocins also form hydrophilic pores in the cell membrane, triggering the amphiphilic C-terminal domain's *α*-helix gate. This can affect the membrane's PMF, causing ion diffusion that leads to the death of the target microbial cell [17]. Subclass IIb bacteriocins permeabilize the cell membrane and decrease intracellular ATP levels in target microbes [13]. Subclass IIc bacteriocins directly interact with the negatively charged cell membrane without requiring receptor molecules [17]. In this case, the N- and C-terminal domains are covalently bound, stabilizing the peptide [41]. These bacteriocins carry two transmembrane segments that facilitate pore formation [26], leading to ion efflux and membrane dissipation, resulting in cell death [17].

Subclass IIIa bacteriocins cause lysis of the target microbial cell wall [37]. Subclass IIIb bacteriocins inhibit glucose absorption, resulting in a carbohydrate deficit that causes cell death [38]. Subclass IIIc bacteriocins compress the cell wall and penetrate the cell nucleus, leading to pore formation [37]. Class IV bacteriocins act by attaching to the cell membrane and facilitating cell lysis [13]. Class V bacteriocins form nonselective pores in the target microbial cell membrane [31].

Bacteriocins can inhibit DNA, RNA, and protein synthesis within cells. Examples include microcin B17 (DNA gyrase), microcin J25 (RNA polymerase), and microcin C7-C51 (aspartyl-tRNA) [26]. Microcin B17 crosses the target microbial cell membrane with the help of a membrane peptide transporter, SbmA, and disrupts DNA replication by preventing supercoiling mediated by DNA gyrase. Microcin J25 suppresses transcription by inhibiting RNA polymerase's secondary channel. Microcin C7-C51 crosses the inner layer of the *Escherichia coli* cell wall with the help of the YejABEF transporter and then inhibits the synthesis of aspartyl-tRNA and mRNA [29]. Certain bacteriocins also have enzymatic activity, such as megacin A-216 (phospholipase), colicin E2 (DNase), and colicin E3 (RNase) [26]. Bacteriocins like thiostrepton, thiazomycin, nocathiacin, and thiopeptides attach to 23S rRNA in the 50S ribosomal subunit and target the ribosome. Bottromycin prevents aminoacyl-tRNA from binding to the 50S ribosome [29]. Other bacteriocins can inhibit the production of essential enzymes, such as colicin E9, leuconocin S, and pediocin JD [42].

## 5. Differences in Characteristics Between Bacteriocin and Antibiotic in Combating Pathogenic Bacteria

Bacteriocins are antibiotic-like compounds that exhibit antimicrobial or therapeutic properties. These compounds are produced by various bacteria and can kill pathogenic bacteria of the same or closely related species. Despite their similarities, bacteriocins differ significantly from antibiotics, particularly concerning their action specificity and the limited composition of their proteins [29]. Bacteriocins are synthesized ribosomally, whereas antibiotics are produced by several enzyme complexes [48]. At low concentrations, bacteriocins are more effective at inhibiting the growth of pathogenic bacteria compared to antibiotics [26]. Bacteriocins also have a narrow spectrum and can only combat the same or closely related bacterial species, while antibiotics have a broad spectrum and can act on bacteria from different genera [48].

In recent years, the prevalence of antibiotic-resistant bacteria has become increasingly common [29]. The Global Antibiotic Resistance and Use Surveillance System reported that in 2020, high levels of antibiotic resistance were observed in 66 countries. The risks posed by antibiotic resistance should not be underestimated [11]. Antibiotic resistance is associated with genetic factors that play a role in transferring resistance to cells, strains, and species [34]. Few new classes of antibiotics have been discovered and explored further to combat antibiotic resistance. Long-term antibiotic use leads to a decrease in effectiveness as pathogenic bacteria can adapt to antibiotics. Another factor contributing to antibiotic resistance is the transmission of resistant bacteria from humans or livestock to humans, which poses a significant public health problem. To date, the discovery and development of new bacteriocins represent a breakthrough in addressing this issue [29]. Additionally, in the food sector, bacteriocins are considered more natural and suitable for use compared to antibiotics. Bacteriocins are not inactivated by enzymes such as pepsin and trypsin, making them easily degradable and not altering the microbiota population in the digestive tract [26].

The differences in characteristics between bacteriocins and antibiotics, including applications, synthesis, size, molecular diversity, spectrum, bioflexibility, host cell immunity, environmental resistance, susceptibility of producing strains to antimicrobial agents, sensitivity to protease enzymes, cytotoxicity, resistance mechanisms in target microbial cells, stability at varying temperatures and pH, and modes of action, are displayed in Table 1.

## 6. Bacteriocin Diversity and the Application as Food Biopreservatives

Bacteriocins produced by lactic acid bacteria have garnered significant attention due to their high biosafety and wide applications in the food industry [38]. In addition to lactic acid bacteria, bacteriocins are also produced by Gram-negative bacteria. The first discovery of bacteriocins was reported in early 1925. This bacteriocin is known as colicin, produced by *Escherichia coli*. The antimicrobial activity of bacteriocins was first discovered in 1928. However, the application of bacteriocins in food products did not occur until 1951. In the 1960s, the discovery of bacteriocins produced by *Lactococcus lactis* subsp. *lactis*, namely, nisin, was reported. This bacteriocin was purified and recognized as a safe food preservative by the Food and Agriculture Organization (FAO)/World Health Organization (WHO) in 1969 [14]. Nisin is commonly used in dairy products, processed cheese, and spreads [48]. To date, nisin has been approved by the FDA for use in more than 48 countries [26, 48]. In addition to nisin, other bacteriocins have been studied and further developed [26].

Bacteriocins are widely studied and applied in the food industry, particularly in processed meat, dairy, vegetables [48], and fruits [12]. These food products are prone to spoilage due to their nutrient content, which supports the growth of spoilage or pathogenic bacteria [38]. For instance, meat is a source of protein, lipids, vitamins, and minerals, which can provide an optimal environment for the growth of bacteria [13], such as *Escherichia coli*, *Campylobacter jejuni*, *Listeria monocytogenes*, *Salmonella enterica*, and *Yersinia enterocolitica* [20]. Additionally, dairy products, such as cheese, can easily be contaminated by bacteria due to their high content of protein, calcium, phosphorus, lactose, and carotene. *Clostridium tyrobutyricum* commonly contaminates cheese and can reduce its quality [6]. Contamination can also occur in vegetable and fruit products during production or processing [12]. Bacteriocins produced by lactic acid bacteria can inhibit the growth of these bacteria during food processing [29]. The diversity of bacteriocins and their applications as food biopreservatives are displayed in Table 2 as follows.

The bacteriocins suitable for application as food biopreservatives are Class I and II bacteriocins [36]. In addition to nisin, a safe bacteriocin for food use is pediocin produced by *Pediococcus pentosaceus* and *Pediococcus acidilactici* [28]. This bacteriocin can inhibit the growth of various Gram-positive bacteria and foodborne pathogens, such as *Listeria monocytogenes* [38]. Furthermore, several bacteriocins that are nearing commercial status for use as food biopreservatives are currently being isolated, characterized, and developed [10]. Examples include carnobacteriocin BM1, carnocyclin A, lacticin 3147 and 481, piscicolin 126 [26], bifidocin B, enterocin, lactatin, lactococcin, lactotin, sakacin, thermophilicin B23 and B67, cerein, plantaricin, subtilin, and thuricin. Cerein, plantaricin, and thuricin can inhibit the growth of *Staphylococcus* sp. Additionally, cerein, plantaricin, and thuricin can each combat *Bacillus* sp., *Listeria* sp., and *Clostridium* sp., respectively [10].

Research related to the exploration of bacteriocins produced by various lactic acid bacteria and their applications as food biopreservatives has been widely conducted. Some of these studies are presented in Table 3.

## 7. Nisin and Pediocin: Commercial Bacteriocin as Food Biopreservatives

Commercial bacteriocins, such as nisin [26] and pediocin, are commonly used as food preservatives [28]. Both bacteriocins are effective as biocontrol agents against *Listeria monocytogenes* in food but are not effective against certain pathogenic bacteria, such as *Clostridium difficile* and *Clostridium tyrobutyricum* [31]. Nisin and pediocin PA-1/AcH are considered safe for use in food systems due to classification as GRAS by the FDA [2]. These peptides are commercially available under the trade names Nisaplin™ by Danisco and ALTA™ 2431 as well as Microgard™ by Quest International [10, 26]. In addition, pediocin PA-1/AcH is commercialized in the form of crude extract obtained from fermentation processes [20]. Nisin market in various developed countries in Europe and North America is rapidly growing. Market estimates with nisin applications in meat, canned, and frozen food products, beverages, bread, confectionery, dairy, poultry, and seafood reached USD 553 million in 2025 [14].

Nisin is a Class I bacteriocin that has been developed since the early 1960s and is produced by *Lactococcus lactis* [14, 26]. Nisin contains 34 amino acids with a molecular mass of 3500 Da and consists of variant A, which contains histidine, and variant Z, which contains asparagine [14]. Both variants share similar molecular patterns and antimicrobial activity. Another variant of nisin is nisin U, produced by *Streptococcus uberis*, which shares 78% similarity with nisin A [34]. Nisin exhibits good stability at cold temperatures. Research by Divsalar et al. [66] showed that nisin remains highly stable at low or refrigerated temperatures, with its antibacterial activity gradually decreasing as the temperature increases from 4°C to 22°C. Based on studies of acute, subchronic, and chronic toxicity, in vitro testing, reproduction, cross-resistance, and sensitization, nisin is safe for human consumption at the acceptable daily intake (ADI) level [14]. Nisin is safe for regular human consumption at a dosage of 2.9 mg/person/day. Additionally, nisin is sensitive to proteolytic enzymes in the stomach. Trypsin can deactivate nisin, thereby not affecting the composition of the microflora in the stomach [34].

Nisin has been used as a food preservative in over 48 countries and is effective in inhibiting the growth of various Gram-positive bacteria found in food, including foodborne pathogens such as *Bacillus* sp., *Clostridium botulinum*, and *Listeria monocytogenes* [26], as well as *Streptococcus* sp. However, nisin does not have a broad inhibitory effect, especially against Gram-negative bacteria [10]. Nisin is primarily used as a preservative in meat and processed meat products. Its application as a preservative in spreadable cheese was approved by the FDA in 1988 [14]. Nisin can inhibit the germination of *Clostridium botulinum* spores in spreadable cheese [25]. Nisin at concentrations of 2000, 4000, and 100 IU/mL effectively inhibits the growth of *Listeria monocytogenes* in cottage cheese, *Bacillus cereus* spores in skim milk, and *Lactobacillus* sp. in kimchi, respectively. Nisin A effectively inhibits the growth of *Listeria monocytogenes* in ricotta cheese for up to 8 weeks. The use of nisin in meat has also been widely reported. At lower levels compared to nitrates, nisin or its combinations can prevent the growth of *Clostridium* sp. in meat [14].

The combination of nisin with other antimicrobial compounds produces a better effect. A formulation of 500 IU/g nisin with 0.6% oregano oil inhibits the growth of *Salmonella enteritidis* in minced meat. Meanwhile, the addition of 0.3 ppm nisin and *p*-cymene suppresses the growth of *Salmonella typhi* in sausages [25]. The combination of nisin with essential oils extracted from grape seeds and green tea also inhibits the growth of *Listeria monocytogenes*. Additionally, nisin with diacetyl can inhibit the growth of *Listeria monocytogenes* and *Enterobacter sakazakii* [16]. The use of two different bacteriocin shows excellent potential in combating target microbes to significantly reduce the minimum inhibitory concentration and lower the inhibitory dose [25]. Nisin and pediocin inhibit the growth of *Listeria monocytogenes* in vegetables [16]. The combination of nisin A with lacticin 3147 can inhibit the growth of *Listeria innocua* in cheese [11]. The combination of the peptide with leucocin F10, pediocin PA-1/AcH, curvaticin 13, and lacticin 481 is effective against *Listeria monocytogenes* [25].

Pediocin is classified as a Class IIa bacteriocin [26] produced by *Pediococcus acidilactici* and *Pediococcus pentosaceus* [28]. This bacteriocin contains aliphatic and aromatic amino acids and has a molecular weight ranging from 2.7 to 17 kDa, consisting of a hydrophilic N-terminal and a hydrophobic C-terminal [33]. Pediocin is encoded by an operon that includes structural genes along with *pedB* (encoding immunity peptide to protect the producing strain), *pedC* (modifying the ABC transporter), and *pedD* (modifying complementary peptides for extracellular translocation) [67]. In food matrices, pediocin is highly active against *Listeria monocytogenes* [26] and *Lactobacillus curvatus* [14]. However, a drawback of pediocin is its instability at room or low temperatures. This issue can be addressed by replacing methionine residues with hydrophobic residues to maintain stability and antimicrobial activity [34].

Pediocin comprises various types, some of which include pediocin PA-1, AcH [14], K10, HW01 [68], A [69], and F [70]. Pediocin PA-1 is produced by *Pediococcus acidilactici* PAC1.0 [71], while pediocin AcH is produced by *Pediococcus acidilactici* H [72]. Meanwhile, pediocin K10 and HW01 are produced by *Pediococcus acidilactici* K10 and HW01, respectively [68]. Pediocin A, produced by *Pediococcus pentosaceus* FBB61 [69], has a molecular weight of 2.7 kDa and is sensitive to heat and proteolytic enzymes [67], such as papain, pepsin, and trypsin [33]. In contrast, pediocin F produced by *Pediococcus acidilactici* F [70] has a molecular weight of 4.5 kDa and is active at pH levels below 6. Pediocin F is sensitive to heat and proteolytic enzymes but resistant to organic solvents [67].

Pediocin is suitable for application in dairy products, such as spreadable cheese, to inhibit the formation of spores from *Bacillus* sp. and *Clostridium* sp. [48]. Pediocin is expressed in *Saccharomyces cerevisiae* to be applied as a preservative for wine and baked food products. In its application on meat, pediocin PA-1/AcH is less active compared to nisin [14]. Additionally, pediocin K10 and HW01 are applied as antibiofilm agents to prevent food contamination by *Salmonella typhimurium*. The presence of *Salmonella typhimurium* in food is related to its ability to form single or multispecies biofilms [68].

## 8. Safety Status and Regulations Related to Bacteriocins

Bacteriocins offer various benefits for food safety and human health compared to synthetic preservatives that are chemically based [29]. Synthetic preservatives, such as sorbic acid, benzoic acid, nitrites, and parabens, are commonly used to inhibit the growth of spoilage bacteria or pathogens in food. The use of these preservatives is associated with issues like asthma, allergies, obesity, etc. Excessive consumption of potassium sorbate and sodium sorbate negatively affects the body's metabolic balance [11]. Another example is sodium nitrate, which is used for preserving sausages and hot dogs and can lead to lung and pancreatic cancer. Propyl gallate used in milk packaging causes inflammation of the prostate gland and tumors in the brain, pancreas, and thyroid gland [14]. Calcium benzoate added to beverages, cereals, and meat reduces aminoglycine levels and inhibits digestive enzyme functions. Sulfur dioxide in potato products, juices, dried fruits, and carbonated beverages can cause asthma, nausea, diarrhea, stomach irritation, skin rashes, and even asthma [15].

Based on stringent safety standards and food ratification accompanied by consumer demand for healthy lifestyles, food preservation techniques using synthetic preservatives, such as acetic acid, benzoic acid, and sorbic acid, have been rejected. This is because these preservation methods have negative impacts on consumers, such as allergies. Moreover, the consumption of synthetic preservatives leads to the formation of carcinogenic products from nitrites, namely, nitrosamines. The shift from synthetic preservatives to biopreservatives is a solution to reduce the negative impacts caused [34]. The increasing consumer demand for healthy food without chemical preservatives encourages the development of biopreservatives that utilize bacteriocins [14]. Furthermore, lactic acid bacteria, which are the source of these bacteriocins, are preferred by consumers because they do not cause cytotoxic effects in the body [19, 34].

Although considered safe and nontoxic, the use of bacteriocins as biopreservatives in food raises concerns among consumers [29]. Some bacteriocins exhibit cytotoxicity when tested using cell culture–based techniques. Bacteriocins can cause cytotoxicity in mammalian cells at concentrations higher than the minimum inhibitory concentration required to protect food from spoilage bacteria or pathogens. This cytotoxic property arises from the binding of bacteriocins to cells or anionic membranes through hydrophobic interactions, thereby directly damaging the cells. For example, semipurified bacteriocin from *Lactiplantibacillus plantarum* ST8SH is cytotoxic at a concentration of 25 *μ*g/mL but not toxic at a concentration of 5 *μ*g/mL. Nisin and pediocin at high concentrations have also been reported to be cytotoxic against Vero cell lines [48]. Research by Cavicchioli et al. [73] reported that nisin exhibited cytotoxicity against Vero, MCF-7, and HepG2 cell lines at concentrations of 13.48, 105.46, and 112.25 *μ*M, respectively. These differences in concentration occur due to variations in the composition of their cell membranes. On the other hand, carnobacteriocin B2 and BM1; colicin E1, E3, E7, and K; enterocin AS-48, DD14, and S37; and plantaricin DM5 showed no or weak cytotoxicity [48].

The unique characteristics of bacteriocins make them potentially applicable to food. However, this needs to be accompanied by good safety aspects [48]. Bacteriocins that can be applied as biopreservatives must be safe for human consumption, have a broad antimicrobial activity spectrum against spoilage bacteria or pathogens in food, be resistant to enzymes, and stable at high temperatures, a wide pH range, and varying salt concentrations. The safety of bacteriocins needs to be further investigated through testing, including cytotoxicity testing in eukaryotic cell lines [34]. This testing aims to determine their cytotoxic effects on eukaryotic cells, their ability to induce apoptosis and hemolysis, growth inhibition [48], in vitro cross-resistance, reproductive disruption, chronic toxicity, and sensitization in animal models. Furthermore, the efficiency of bacteriocin use and its pharmacodynamics should be characterized more thoroughly [34].

Starter cultures producing bacteriocins used as food additives must have GRAS status as specified in food safety regulatory guidelines [23, 34]. GRAS status is typically granted to bacteriocinogenic cultures found in fermented foods that have long been consumed by humans. However, the application of bacterial cultures as additives or preservatives in nonfermented foods still requires FDA approval. The strains used should not affect the organoleptic quality of the food. Additionally, the use of pure bacteriocins and genetically engineered products must follow the safety evaluation guidelines for new preservatives set by the FDA. The genetic materials and vectors used must originate from organisms considered safe in food systems. All allergenic, pathogenic, and toxic properties must be completely eliminated and verified through animal studies and in vitro tests. The nutritional composition of the food must also be evaluated [34]. This is done so that bacteriocins can be marketed and authorized for use as food biopreservatives by the FDA and WHO [48]. Approval from these organizations requires various documentation, including quantification, manufacturing processes, toxicology data, and standardization tests [34].

## 9. Challenges

Bacteriocins have proven to be antimicrobial and have great potential for application as food biopreservatives [48]. However, various challenges are associated with their use. Achieving optimal bacteriocin production is a major challenge [13]. This relates to the slow production of bacteriocins and the growth rate or increase in biomass of the producing bacterial cells at the later stages of their life cycle [29]. The growth of these bacteria requires specific conditions and nutritional compositions [34]. Factors such as the presence of acetic acid, microbial cell density, availability of nutrients (amino acids, carbohydrates, minerals, proteins, organic nitrogen sources, and vitamins), temperature, and pH of the culture medium must be considered to support optimal bacteriocin production [31, 74]. Additionally, some bacteriocin-producing bacteria are unable to produce sufficient amounts of bacteriocins and are ineffective in protecting food [34].

High costs associated with the isolation, production, and purification of bacteriocins pose a challenge to their application as food biopreservatives. Future efforts should focus on developing cost-effective bacteriocin purification methods without compromising quality and efficiency [13]. Susceptibility to extreme pH and high temperatures [34], uneven distribution, and suboptimal binding to food matrices remain limitations to this day [38]. The effectiveness of bacteriocins applied to food needs to be further researched and developed. Bacteriocins produced may not be effective or stable when applied to various food systems due to the significant influence of the physical conditions and chemical compositions of food on bacteriocin activity. Some bacteriocins work optimally when used in the appropriate food conditions or matrices [34].

The development of bacteriocin resistance can jeopardize their role as food biopreservatives, presenting a major concern in the food sector [29]. Manipulation of surface charge and membrane fluidity of the target microbial cells results in decreased bacteriocin effectiveness, leading to bacteriocin resistance [75]. Bacteriocin resistance is associated with physiological changes in the target microbial cell membranes. Understanding the mechanisms of bacteriocin function and degradation processes is essential to avoid these resistance issues and to ensure the long-term effectiveness of their use [34]. Additionally, their narrow spectrum of activity presents an obstacle for bacteriocins. Some bacteriocins are only effective against Gram-positive bacteria and are less potent against Gram-negative bacteria [41]. The cell membrane of Gram-negative bacteria consists of an inner membrane, peptidoglycan, cell wall, and outer membrane. The protective presence of the outer membrane prevents bacteriocins from interacting with lipid II precursors, thereby limiting their use as food biopreservatives [11].

At low doses, bacteriocins effectively kill vegetative bacterial cells but are unable to destroy endospores, requiring higher doses [29]. The use of excessive doses of bacteriocins can affect the sensory quality of food [11] and lead to nonspecific bactericidal effects that are harmful to the body. This can be mitigated by using a combination of different bacteriocins or combining bacteriocins with other antimicrobial compounds [25]. The potential of bacteriocins can also be enhanced through biotechnological approaches. Bacteriocins can be biologically engineered at specific amino acid residues to produce physicochemical properties that can enhance their expression and activity against target microbes in food. This effort is expected to improve the effectiveness and stability of bacteriocins as antimicrobials, reduce production and purification costs, and minimize the negative impacts of chemical preservatives [34].

So far, the use of several potential bacteriocins has not yet received approval for application and commercialization as food preservatives in several countries. Cytotoxicity is a fundamental factor behind this. Some bacteriocins exhibit cytotoxic properties when tested on mammalian or eukaryotic cells. High concentrations of bacteriocins in food can pose health risks. Further testing is required regarding the safety of bacteriocins, starting from their cytotoxic effects, apoptotic and hemolytic abilities, growth inhibition [48], in vitro cross-resistance, reproductive disruption, chronic toxicity, and sensitization. Additionally, the application of bacteriocins is hindered by food safety regulations. Bacteriocins intended for commercialization must meet applicable food safety standards set by the FDA [34]. Bacteriocin-producing bacteria must also have GRAS status to ensure they are safe and suitable for consumption [23, 34].

## 10. Conclusion and Future Prospects

Food safety is a critical issue affecting health and economic aspects for communities worldwide. In this regard, bacteriocins attract significant attention in the food industry. Bacteriocins produced by lactic acid bacteria have become subjects of intensive study and development. Their unique characteristics, such as pH tolerance, thermal stability, nontoxicity, and ease of degradation by proteolytic enzymes, make them promising for application as biopreservatives or natural preservatives in various countries. Nisin and pediocin are commercial bacteriocins currently used in various food products, including eggs, milk, meat, fruits, vegetables, and processed food items. Various bacteriocins have been identified, but there has been limited progress in obtaining approval from government agencies for their industrial applications. This relates to their safety. Toxicity testing or in vivo research is necessary to evaluate the biological activity and safety of bacteriocins, including acute and subacute toxicity, immunogenicity, absorption, and side effects. Furthermore, there is a need to optimize the production and extraction processes of bacteriocins to achieve maximum results. Future research should focus on discovering, purifying, and developing new bacteriocins with promising properties for application as food biopreservatives. Additionally, the stability and antimicrobial activity of bacteriocins need to be enhanced to combat Gram-negative bacteria, particularly in food matrices. Further research is also required to maximize the potential of bacteriocins through biotechnological or genetic engineering approaches and by combining applications with other antimicrobial compounds. All these efforts aim to address the challenges of antibiotic resistance while replacing the roles of chemical preservatives to ensure better food safety and human health.

## Figures and Tables

**Table 1:** Differences in characteristics between bacteriocin and antibiotic in combating pathogenic bacteria.

Characteristics	Bacteriocin	Antibiotic	Reference
Application	Food	Clinical	Ghosh et al. [14]
Synthesis	Ribosomal	Several enzyme complexes	Ghosh et al. [14]; Negash and Tsehai [26]
Size	15–80 kDa	0.3–0.5 kDa	Hahn-Löbmann et al. [49]
Molecular diversity	> 800	∼150	Hols et al. [50]
Spectrum	Narrow, mostly intraspecific selectivity	Wide	Hahn-Löbmann et al. [49]; Negash and Tsehai [26]; Pircalabioru et al. [47]
Bioflexibility (engineering)	High	Low	Hols et al. [50]
Host cell immunity	Yes	No	Ghosh et al. [14]
Environmental resilience	Low	High	Hols et al. [50]
Vulnerability of producer strains to antimicrobial agents	Not vulnerable	Vulnerable	Gholami et al. [43]
Sensitivity to protease enzymes	Sensitive	Less sensitive	Pircalabioru et al. [47]
Cytotoxicity	Low	High	Hols et al. [50]
Mechanisms of target microbial cell resistance	Adapting by influencing the content of the cell membrane, which exhibits resistant properties	Transferring genetically determined factors that affect different sites depending on the mode of action of each antibiotic	Ghosh et al. [14]
Stability at temperature and pH	High for bacteriocin Classes I and II	Low	Hols et al. [50]
Mode of action	Forming pores and inhibiting the synthesis of the target microbial cell wall	Targeting the cell membrane or intracellularly of the target microbial cell	Ghosh et al. [14]

**Table 2:** Diversity of bacteriocin and applications as food biopreservatives.

Type of food	Food applications	Type of bacteriocin	Bacteria producers	Spoilage or pathogenic bacteria	Reference
Meat products	Meat	Sukacin	*Latilactobacillus sakei*	*Listeria monocytogenes*	Saraoui et al. [51]
	Meat	Mycocin	*Debaryomyces hansenii* DSMZ70238	*Listeria monocytogenes*	Elsayed, Farid, and El-Enshasy [52]
	Meat	Bacteriocin 7293A and 7293B	*Weissella hellenica* BCC 7239	*Aeromonas hydrophila*, *Escherichia coli,* and *Salmonella typhimurium*	da Costa et al. [53]
	Chicken and beef	Sakacin P	*Latilactobacillus curvatus* CWBI-B28	*Listeria monocytogenes*	Bhattacharya et al. [13]
	Ground Turkey	Enterocin BacFL31	*Enterococcus faecium* FL31	*Listeria monocytogenes*, *Salmonella typhimurium*, and *Staphylococcus aureus*	Bhattacharya et al. [13]
	Pork	Pediocin PA-1/AcH	*Pediococcus acidilactici* MCH14	*Listeria monocytogenes*	Bhattacharya et al. [13]
	Beef and chicken	Bacteriocin BacTN635	*Lactiplantibacillus plantarum* TN635	*Listeria* sp.	Radaic, de Jesus, and Kapila [54]
	Meat and sausages	Nisin	*Lactococcus lactis*	*Listeria monocytogenes* and *Staphylococcus aureus*	Silva, Silva, and Ribeiro [55]
	Pork sausages	Pediocin PA-1/AcH	*Pediococcus pentosaceus* BCC 3772	*Listeria monocytogenes*	Bhattacharya et al. [13]
	Fermented meat sausages	Pediocin bacHA-6111-2	*Pediococcus acidilactici* HA-6111-2	*Listeria innocua*	Castro et al. [56]
	Fermented dry sausages	Enterocin A and B	*Enterococcus faecium* CTC492	*Listeria innocua*	Vishwanatha et al. [33]

Dairy products	Skim milk	Nisin	*Lactococcus lactis*	*Bacillus cereus*	Ghosh et al. [14]
	Milk	Leucocin A	*Leuconostoc gelidum* UAL187	*Listeria monocytogenes*	Balay, Gänzle, and McMullen [57]
	Milk	Bacteriocin CAMT2	*Bacillus amyloliquefaciens* ZJHD3-06	*Vibrio parahaemolyticus*, *Staphylococcus aureus*, *Listeria monocytogenes*, and *Escherichia coli*	Wu et al. [58]
	Milk	Leucocin K7	*Leuconostoc mesenteroides* K7	*Listeria monocytogenes*	Vishwanatha et al. [33]
	Cheese	Lacticin 3147	*Lactococcus lactis* subsp. *lactis* DPC3147	*Listeria monocytogenes*	Lahiri et al. [34]
	Cheese	Nisin	*Lactococcus lactis*	*Bacillus cereus* and *Bacillus subtilis*	Silva, Silva, and Ribeiro [55]
	Cheddar cheese	Nisin	*Lactococcus lactis*	*Listeria monocytogenes* and *Staphylococcus aureus*	Silva, Silva, and Ribeiro [55]
	Raw soft cheese and skim milk	Enterocin AS-48	*Enterococcus faecalis* A-48-32	*Staphylococcus aureus*	Silva, Silva, and Ribeiro [55]
	Spreadable cheese	Nisin	*Lactococcus lactis*	*Listeria monocytogenes*, *Staphylococcus aureus*, and *Clostridium sporogenes*	Banerjee et al. [29]
	Cottage and ricotta cheese	Nisin	*Lactococcus lactis*	*Listeria monocytogenes*	Ghosh et al. [14]
	Cottage cheese, cream, and cheese sauce	Pediocin PA-1/AcH	*Pediococcus pentosaceus*	*Listeria monocytogenes*	Silva, Silva, and Ribeiro [55]
	Formula milk, yogurt, and cottage cheese	Lacticin 3147	*Lactococcus lactis* subsp. *lactis* DPC3147	*Listeria* sp. and *Bacillus* sp.	Silva, Silva, and Ribeiro [55]
	Skim milk and yogurt	Enterocin CCM 4231	*Enterococcus faecium* CCM 4231	*Listeria monocytogenes* and *Staphylococcus aureus*	Silva, Silva, and Ribeiro [55]
	Skim milk and whole milk	Lactococcin BZ	*Lactococcus lactis* subsp. *lactis* BZ	*Listeria monocytogenes*	Silva, Silva, and Ribeiro [55]

Vegetable product	Vegetable soup and porridge	Enterocin AS-48	*Enterococcus faecalis* A-48-32	*Bacillus cereus*, *Bacillus macroides*, and *Paenibacillus* sp.	Vishwanatha et al. [33]
	Vegetable porridge (zucchini)	Enterocin EJ97	*Enterococcus faecalis* EJ97	*Bacillus macroides* and *Bacillus macroccanus*	Vishwanatha et al. [33]

Fruit product	Fruit	Colicin GRN 676 and GRN 593	*Escherichia coli*	*Escherichia coli*, *Pseudomonas aeruginosa*, and *Salmonella* sp.	Hahn-Löbmann et al. [49]
	Canned fruit	Enterocin AS-48	*Enterococcus faecalis* A-48-32	*Bacillus coagulans*	Vishwanatha et al. [33]
	Fruit juice	Enterocin AS-48	*Enterococcus faecalis* A-48-32	*Alicyclobacillus acidoterrestris*	Vishwanatha et al. [33]
	Apple juice	Enterocin AS-48	*Enterococcus faecalis* A-48-32	*Bacillus licheniformis*	Vishwanatha et al. [33]

**Table 3:** Studies of various bacteriocins and applications as food biopreservatives.

Type of bacteriocin	Class of bacteriocin	Bacteria producers	Food applications	Effects on food	Reference
Enterocin BacFL31	II	*Enterococcus faecium* FL31	Ground beef	Enterocin BacFL31 (200 AU/g) combined with an aqueous extract of red onion skin (1.56 ± 0.3 mg/mL) in ground beef. The effect includes limiting microbial damage, reducing thiobarbituric acid reactive substances (TBARS), slowing the accumulation of metmyoglobin (MetMb) and carbonyl groups, delaying the loss of sulfhydryl proteins, preventing pathogen proliferation, decreasing primary and secondary lipid oxidation, reducing protein oxidation, and significantly enhancing the sensory attributes of ground beef during 14 days of storage at 4°C	Mtibaa et al. [59]
Fermencin SA715	I	*Lactobacillus fermentum* GA715	Fresh bananas	Fermencin SA715 can enhance the shelf life and microbiological safety of fresh bananas. The total bacterial count on bananas treated with fermencin is lower (2.36 × 10^3^ and 2.14 × 10^3^) compared to the control (4 × 10^7^ and 3.7 × 10^5^) at room temperature and cold storage, respectively. Fermencin also extends the storage life of bananas by 6 and 9 days, at room temperature and cold storage	Wayah and Philip [60]
Leucocin A	II	*Leuconostoc gelidum* UAL187	Milk, fresh beef, and sausages	The activity of leucocin A against *Listeria monocytogenes* remains stable in milk, fresh beef, and sausages	Balay, Gänzle, and McMullen [57]
Leucocin B	II	*Leuconostoc carnosum* Ta11a	Meat and meat products	The activity of leucocin B does not affect the pH and taste of meat	Hwang et al. [61]
Leucocin C	II	*Leuconostoc carnosum* 4010	Raw chicken breast	A beer containing leucocin C is used as a solution for marinating chicken breast pieces. The URA3 auxotrophic strain from the probiotic yeast *Saccharomyces boulardii* CNCM I-745 is also used as a host to express the lecC gene encoding leucocin C. The beer inhibits the growth of *Listeria monocytogenes* by approximately 1.6 logs from (2.2 ± 0.6) × 10^7^ CFU/g on day 24 and 2.2 logs from (1.8 ± 0.3) × 10^5^ CFU/g on day 38 of storage	Li et al. [62]
Nisin A	I	*Lactococcus lactis*	Cheddar cheese	A nisin formula encapsulated with 1% alginate and 0.5% corn starch reduces the amount of *Clostridium tyrobutyricum* by 1.4 logs after 1 week of incubation at 4°C. From the second to the fourth week, *Clostridium tyrobutyricum* was not detected in cheddar cheese	Hassan et al. [1]
Nisin Z	I	*Lactococcus lactis* subsp. *lactis* I8-7-3 LC 113942	Bigeye snapper (*Lutjanus lineolatus*) and tiger prawn (*Penaeus monodon*)	Nisin (69.4 AU/cm^2^) combined with thermoplastic starch (TPS) or polybutylene adipate terephthalate (PBAT) can reduce the amount of *Salmonella typhimurium* ATCC 14028 by 7 log10 CFU/g and 3.5 log10 CFU/g after 14 and 28 days at 4°C (bigeye snapper and tiger prawn). This combination also decreases *Vibrio parahaemolyticus* by 5.8 log10 CFU/g after 14 days as well as 4.2 log10 CFU/g and 7.1 log10 CFU/g after 28 days at 4°C	Pattanayaiying et al. [63]
Pediocin bacHA-6111-2	I	*Pediococcus acidilactici* HA-6111-2	Alheiras (traditional Portuguese fermented meat)	Pediocin combined with pressure treatment (300 MPa for 5 min) inhibits the growth of *Listeria innocua* in Alheiras during storage for 60 days at 4°C. The amount of *Listeria innocua* is reduced and maintained below the detection limit (< 2 log CFU g^−1^) on the first day of storage	Castro et al. [56]
Plantaricin BM-1	II	*Lactiplantibacillus plantarum* BM-1	Fresh pork	Plantaricin BM-1 (20,480 AU/mL) combined with polyvinylidene chloride (PVDC) film. This film inhibits the growth of *Listeria monocytogenes* and significantly reduces the aerobic bacteria count by 1.5 log10 CFUg^−1^ after 7 days of storage at 4°C. Total volatile basic nitrogen (TVB-N) and pH decreased significantly compared to the control	Xie et al. [64]
Sakacin G	II	*Lactobacillus curvatus* ACU-1	Cooked sausages	The cell-free supernatant (CFS) from *Lactobacillus curvatus* ACU-1 strain containing sakacin G is effective in controlling *Listeria* sp. and other bacteria causing spoilage in cooked sausages	Rivas and Garro [65]

## Data Availability

The data that support the findings of this study are available on request from the corresponding author. The data are not publicly available due to privacy or ethical restrictions.
